# Clitoral preputial edema can be mistaken for clitoromegaly: a clinical analysis of ten cases

**DOI:** 10.3389/fendo.2023.1175611

**Published:** 2023-07-07

**Authors:** Marie Mitani-Konno, Reiko Saito, Hiroko Narumi-Wakayama, Yuki Sakai, Shuichi Suzuki, Hiroyuki Satoh, Yukihiro Hasegawa

**Affiliations:** ^1^ Division of Endocrinology and Metabolism, Tokyo Metropolitan Children’s Medical Center, Tokyo, Japan; ^2^ Department of Pediatrics, Keio University School of Medicine, Tokyo, Japan; ^3^ Department of Pediatrics, National Defense Medical College Hospital, Saitama, Japan; ^4^ Department of Urology, Tokyo Metropolitan Children’s Medical Center, Tokyo, Japan

**Keywords:** clitoral preputial edema, clitoromegaly, difference of sexual development, external genitalia, neonates

## Abstract

**Background and objectives:**

We herein reported ten, female neonates with transient clitoral preputial edema, which was mistaken for clitoromegaly. Although it is well known that the clitoris is prominent in premature, female neonates, there are as of yet no reports of clitoral preputial edema in full-term neonates. The present study was conducted to clarify the clinical course of clitoral preputial edema.

**Methods:**

Seventeen, Japanese patients aged < 6 months with suspected clitoromegaly were enrolled, and their clinical course was analyzed retrospectively. Clitoral preputial edema was defined by 1) a normal clitoral glans despite edema; and 2) the absence of established differences of sexual development, such as 21-hydroxylase deficiency.

**Results:**

Ten of the 17 patients with suspected clitoromegaly had clitoral preputial edema; eight of the ten patients were full-term, and the remaining two were preterm neonates. The median age at the first visit was 8 days. Edema of the labia minora and labia majora, rugosity of the labia majora, and hymenal polyps often accompanied the clitoral preputial edema. Seven patients were examined at our division during the neonatal period, and three patients were examined in the post-neonatal period. Age at reduction of the clitoral width to < 7 mm ranged from 8 to 74 days in four of the seven neonatal patients. In the three post-neonatal patients, age to reduction in the clitoral width ranged from 107 to 243 days.

**Conclusions:**

Transient clitoral preputial edema is often mistaken for clitoromegaly. The key to diagnosing clitoral preputial edema lies in its characteristic appearance and improvement course.

## Introduction

The clitoris can appear enlarged in premature, female neonates because of underdeveloped labia majora (1), absence of vulval fat, and labial or clitoral preputial edema (2). In 44% of full-term neonates, the clitoris may seem enlarged because the labia majora are not large enough to cover it (3). However, to date, no full-term neonates with transient, clitoral preputial edema mimicking clitoromegaly have been reported.

Clitoromegaly is one of the important signs indicating differences of sexual development (DSD) and should therefore be carefully examined. Evaluation must be carried out at a center with an experienced, multidisciplinary team (4). If sex assignment is a consideration, the decision to perform it should await an expert’s evaluation. Although early postnatal transfer or delayed sex assignment is occasionally necessary in patients with clitoromegaly, the possibility of an early transfer or delayed sex assignment may cause significant emotional stress to the patient’s family (4). Hence, the clitoris should be carefully examined to determine if in fact it is pathologically enlarged or just prominent (5, 6).

Clitoromegaly is diagnosed on the basis of the clitoral width and length (4), including the thickness of the clitoral prepuce. Enlarged clitoral width and length caused by preputial edema in the absence of glans enlargement should not be diagnosed as clitoromegaly. The present study was conducted to clarify the clinical course of clitoral preputial edema.

## Materials and methods

Seventeen Japanese patients with suspected clitoromegaly aged < 6 months were retrospectively analyzed between 2010 and 2021. The final diagnosis and clinical and laboratory findings were extracted from the medical records. Clitoral preputial edema was defined by 1) the presence of a normal clitoral glans despite edema; and 2) the absence of DSD, such as 21-hydroxylase deficiency (21OHD). A clitoral width and length of 7 mm or less were considered normal (7). Tests to rule out DSD, such as chromosomal analysis, abdominopelvic ultrasound imaging, and serum and urinary hormone analyses, were conducted at the discretion of the treating physician. Ethics approval was obtained from the ethics committee at Tokyo Metropolitan Children’s Medical Center (2021b-33). Because the data were collected retrospectively from the patients’ medical records, the ethics committee waived the requirement for written informed consent, and the opt-out consent approach was adopted. Written comprehensive informed consent for the collection and publication of the data was obtained at the first visit of the patients’ parents to our hospital.

## Results

In the total cohort, the final diagnoses were transient clitoral preputial edema for ten, 21OHD for four, persistent cloaca for one, 46,XY DSD for one, and idiopathic clitoromegaly for one patient. All the patients received female sex assignment.

**Table 1** shows the clinical data on the ten patients with clitoral preputial edema. Eight of these were full-term, and the remaining two were preterm. None had perinatal asphyxia. In one patient (Patient 2), clitoral enlargement was first noted during a fetal ultrasound examination at 28 weeks’ gestation. Fetal ultrasound found no abnormalities in the other patients. The median age at the first visit was 8 days. Seven patients were examined during the neonatal period, and three patients were examined during the post-neonatal period.

**Table 1:** External genitalia findings in the ten patients with clitoral preputial edema.

Patient No.	Age at first visit ^a^ (days)	Gestational age at delivery (weeks)	Clitoral width ^b^ (mm)	Clitoral length ^b^ (mm)	Labia minora edema ^c^	Labia majora edema ^c^	Labia majora rugosity ^c^	Hymenal polyps ^c^	Age at normalization of clitoral width (days)	Age at normalization of edema in entire external genitalia (days)
1	1	33	12	13	+	+	N/A	+	60	179
2	4	40	8	10	+	+	+	+	12	104
3	5	36	N/A	N/A	+	−	−	+	N/A	45
4	7	40	10	8	N/A	N/A	−	N/A	N/A	189
5	8	40	7	N/A	+	+	+	+	8	161
6	8	37	N/A	N/A	+	−	−	+	N/A	125
7	19	39	N/A	8	+	+	N/A	N/A	74	200
8	37	39	10	12	N/A	N/A	+	−	243	3-year-old
9	41	39	7	10	N/A	N/A	N/A	N/A	107	N/A
10	65	37	7	9	−	−	+	−	143	143

N/A, not available.

a Patients 1 - 7 were examined during the neonatal period. Patients 8 - 10 were examined after the neonatal period.

b Measured at the first visit.

c Findings made at the first visit to the follow-up are indicated by +. Other findings are indicated by -.

**Figure 1:**
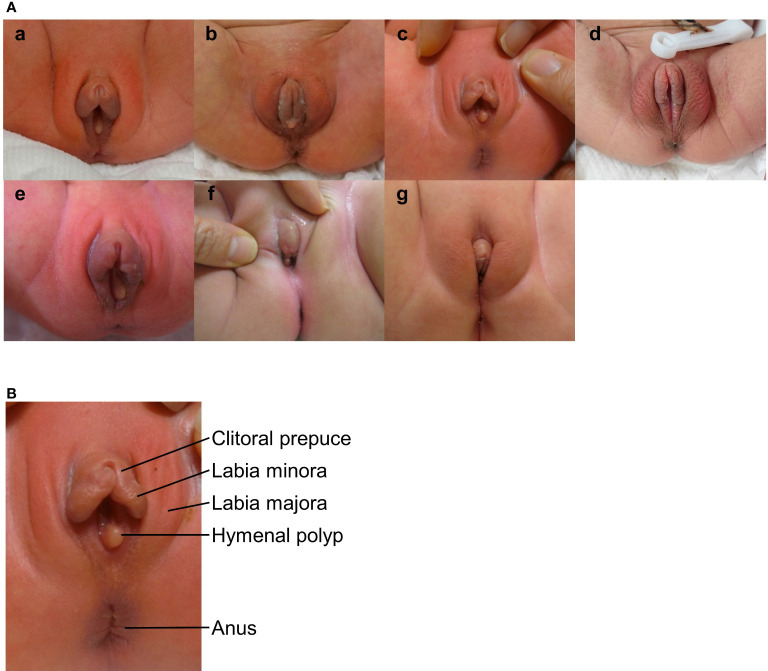
**(A)** External genitalia in clitoral preputial edema. a) Patient 1 at age 1 day; b) Patient 2 at age 4 days; c) Patient 3 at age 5 days; d) Patient 5 at age 1 day; e) Patient 6 at age 8 days; f) Patient 8 at age 77 days; g) Patient 10 at age 65 days **(B)** Parts of the external genitalia with clitoral preputial edema (Patient 3). The clitoral preputial edema was accompanied by edema of the labia minora and hymenal polyps.

**Figures 1A, B** show photographs of the patients’ external genitalia. The median width and length of the clitoris, including the prepuce, was 8 mm and 10 mm, respectively (**Table 1**). Of the seven, neonatal patients, six had the edema of the labia minora. In these patients, the labia minora appeared continuous with the clitoral prepuce. As well, four patients had edema of the labia majora, and hymenal polyps were found in at least five of the seven neonatal patients. Edema was not found in the labia minora or labia majora of the three post-neonatal patients, but rugosity of the labia majora indicating previous edema was found in two patients. One patient (Patient 5) had neonatal menstruation on postnatal days 3 through 7.

21OHD, the most common form of 46,XX DSD, was ruled out in all ten patients with clitoral preputial edema on the basis of the external genital appearance and the results of several tests as follows. None of the patients had the pigmentation characteristic of 21OHD, and the external urethral orifice and vaginal orifice were distinct. The anogenital ratio was normal in the patients with clitoral preputial edema in whom it was measured. No mass was palpable in the labia majora or the inguinal region. G-banded karyotype and/or fluorescence *in situ* hybridization of *SRY* was done in five patients. Abdominopelvic ultrasound imaging was done in all ten patients. Newborn screening for 17-hydroxyprogesterone (17OHP) was done in all ten patients. The serum adrenal hormone profile other than 17OHP was analyzed in nine patients, and urinary steroid profiling was done in seven patients. Anti-Müllerian hormone was measured in two patients. None of these tests found any abnormality.

**Figure 2:**
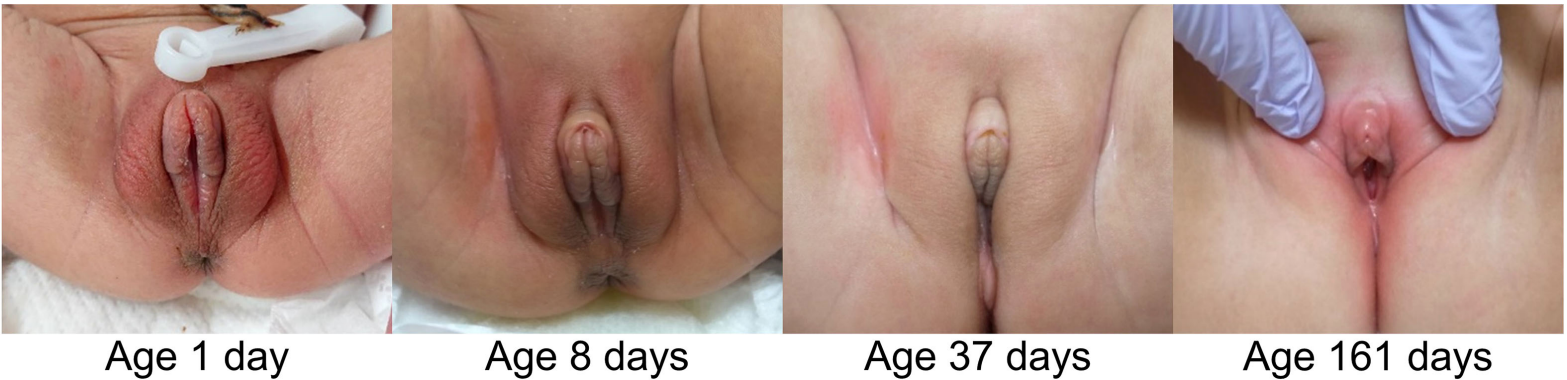
Changes in external genital appearance in Patient 5. The clitoral width improved by age 8 days, and sex assignment was done by age 12 days after several tests. Edema of the entire external genitalia resolved by age 161 days.

The clitoral preputial edema in all the ten patients improved. In the seven neonatal patients in particular, the edema showed improvement daily or weekly. In four of the seven neonatal patients, age at reduction of the clitoral width to < 7 mm ranged from 8 to 74 days. **Figure 2** shows the improvement in the appearance of the external genitalia in Patient 5, who was representative of the entire cohort. The post-neonatal patients required more time until complete normalization of the clitoral appearance and regression of the edema in the entire external genitalia. The last examination date for all the ten patients ranged from age 3 months to 3 years.

Sex was not assigned to six of the seven neonatal patients by their previous physician at birth because DSD was suspected. Sex assignment of these patients was done promptly after they were referred to our hospital, as the birth certificate must be submitted by age 13 days according to Japanese law.

Clitoral preputial edema differs from other diseases in appearance. The Supplemental Figure shows photographs of the external genitalia of patients with a diagnosis other than clitoral preputial edema. Four patients with 21OHD had labioscrotal hyperpigmentation, and three of the four had urogenital sinus with a single, perineal orifice. Moreover, the clitoral length was significantly greater in the patients with 21OHD than in the patients with clitoral preputial edema (median clitoral length: 20 vs. 10 mm; p = 0.017) (**Table 2**). In the patients with persistent cloaca or 46,XY DSD, the external urethral orifice and vaginal orifice were indistinct. In the patient with idiopathic clitoromegaly, the clitoral glans was enlarged, extruded from the prepuce, and became erect during palpation, as described previously in a report of idiopathic clitoromegaly in premature neonates (8). 21OHD was able to be distinguished from clitoral preputial edema by appearance, but additional blood tests and imaging studies were performed for the final diagnosis. The diagnosis of 21OHD was made in all four patients on the basis of elevated serum 17OHP levels on newborn screening and a urinary steroid profile characteristic of this disorder.

**Table 2:** Clitoral width and length in clitoral preputial edema and 21-hydroxylase deficiency.

	Clitoral preputial edema		21-hydroxylase deficiency		p value^#^
	n	Median (mm) (Minimum-maximum)	n	Median (mm) (Minimum-maximum)	
Clitoral width ^*^	7	10 (7–13)	4	10 (8–10)	0.412
Clitoral length ^*^	7	10 (8–13)	3	20 (20–23)	0.017

*Clitoral width and length included the prepuce.

# Mann-Whitney U test.

## Discussion

The findings of the present study suggested that clitoral preputial edema is often mistaken for clitoromegaly, and 59% of the patients with suspected clitoromegaly eventually received a diagnosis of clitoral preputial edema. Since clitoral preputial edema eventually improves, it may be considered a transient finding.

Few studies have described the morphology or edema of the neonatal clitoral prepuce. In the present cohort, clitoral preputial edema was accompanied by edema of the labia minora and majora or rugosity of the labia majora. In their study of the morphology of the clitoral prepuce in subjects aged 0 to 16 years (9), Brodie et al. described the labia minora and clitoral prepuce as distinct structures. However, in the present study, these two structures appeared to be continuous, probably owing to the edema. When clitoral preputial edema is accompanied by edema of the labia minora, the clitoris may resemble a penis whereas edema or rugosity may cause the labia majora to resemble a scrotum. Such findings may easily cause a clinician to mistake clitoral preputial edema for clitoromegaly and refer the patient to DSD experts.

Clinicians should take due note that clitoral preputial edema mimics clitoromegaly but is not pathological; patients with this condition can be discharged earlier and be assigned female sex without delay. However, any infant with atypical external genitalia should be assumed to have DSD until proven otherwise (10). A careful physical examination, follow-up, and necessary testing, as described below, should be performed.

The key to diagnosing clitoral preputial edema is its characteristic findings and its improvement over time. First, the clitoral preputial edema is accompanied by edema in other parts of the external genitalia, but no other abnormalities are present. The following are the minimal criteria for suspecting clitoral preputial edema: 1) absence of an enlarged or exposed clitoral glans; 2) absence of pigmentation; 3) a distinct, external urethral orifice and vaginal orifice; 4) a normal anogenital ratio; and 5) absence of a palpable mass in the labia majora or the inguinal region. In the present study, clitoral preputial edema was able to be differentiated from other diseases on the basis of these criteria. Although a significant difference in clitoral length between clitoral preputial edema and 21OHD was observed, the number of subjects in this study was small, and patients with mild 21OHD symptoms are present.

Second, the probability of clitoral preputial edema increases if the clitoral size normalizes over time. In neonatal patients, the clitoral width normalizes by age 2 months at the latest, and edema of the entire external genitalia improves by age 6 months. Clitoromegaly stemming from mild, classical 21OHD might be difficult to diagnose at birth but will worsen if left untreated (3). Normalization of the clitoral size and regression of edema support the diagnosis of clitoral preputial edema.

Ultrasound imaging and adrenal hormone tests may be considered (8) if distinguishing clitoral preputial edema from clitoromegaly is difficult. Ultrasound imaging is needed to evaluate for the presence of internal female genitalia and the absence of inguinal gonads. While ultrasound imaging is simple and non-invasive, it may not fully visualize the internal genitalia or gonads. An adrenal hormone test may be needed to rule out 21OHD, and 17OHP is recommended for newborn screening for 21OHD in the clinical practice guidelines (11).

The mechanism underlying clitoral preputial edema is not fully understood. It is hypothesized that maternal estrogen plays a role. First, the patients with clitoral preputial edema in the present study were more frequently associated with hymenal polyps, which reportedly result from maternal estrogen exposure (12, 13). Among normal, female neonates, the incidence of hymenal polyps was reportedly 6-13% (14, 15) whereas 71% of the patients with clitoral preputial edema in the present study had hymenal polyps. No physical findings suggestive of estrogen exposure, such as thelarche or pigmented nipples, were observed in any of the patients other than Patient 5, who had neonatal menstruation. Second, estrogens increase capillary permeability and sodium and water retention (16). The thin skin of the clitoris is also thought to be more susceptible to edema formation.

Indeed, the clinical findings support the hypothesis for maternal estrogen involvement. Patient 2 had an enlarged clitoris at 28 weeks’ gestation, and maternal estrogen was elevated in the third trimester (17). The clitoral preputial edema in the neonatal patients improved daily or weekly in keeping with the rapid drop in the maternal estrogen level during the first, few postnatal days (18). Thus, if maternal estrogen is involved in the development of clitoral preputial edema, its absence might improve the symptoms.

The cause of the persistent edema in the three, post-neonatal patients was difficult to identify. Maternal estrogen was unlikely to be the main cause. Clearly, the mechanism underlying edema may differ between neonatal and post-neonatal patients, and some factor besides maternal estrogen is likely to be responsible for the persistence of the symptoms in the latter. Further research is needed to explain the mechanisms giving rise to clitoral preputial edema in infants.

## Conclusion

Transient edema of the clitoral prepuce in neonates is often mistaken for clitoromegaly. However, clitoral preputial edema may be a normal, albeit underreported, condition. Clitoral preputial edema improves over a few weeks to months and may be accompanied by edema in other parts of the external genitalia. It is crucial to rule out DSD in neonates with atypical external genitalia; a diagnosis of clitoral preputial edema can reduce the burden and stress on patients and their family alike caused by needless tests.

## Data availability statement

The original contributions presented in the study are included in the article/**Supplementary Material**. Further inquiries can be directed to the corresponding author.

## Ethics statement

The studies involving human participants were reviewed and approved by Tokyo Metropolitan Children’s Medical Center’s ethical review board. Written informed consent to participate in this study was provided by the participants’ legal guardian/next of kin.

## Author contributions

MM-K examined the patients, collected the clinical cases, drafted the initial manuscript, and revised the manuscript. RS, HN-W, YS, and SS examined the patients. HS supervised the final diagnosis. YH conceptualized and designed the study and critically reviewed the manuscript for important intellectual content. All the authors approved the final manuscript as submitted and agree to be accountable for all aspects of the work.
