# Supplementary material for: Clitoral preputial edema can be mistaken for clitoromegaly: a clinical analysis of ten cases
Source: Front Endocrinol (Lausanne). 2023 Jul 7;14:1175611. doi: 10.3389/fendo.2023.1175611 (PMC10360117; doi:10.3389/fendo.2023.1175611)
Supplement: Supplementary file 1 [file Image_1.pdf]

*Supplementary Material*

# Clitoral preputial edema can be mistaken for clitoromegaly: a clinical analysis of ten cases

**Marie Mitani-Konno*, Reiko Saito, Hiroko Narumi-Wakayama, Yuki Sakai, Shuichi Suzuki, Hiroyuki Satoh, Yukihiro Hasegawa.**

**Correspondence*:** Marie Mitani-Konno: marymitani@yahoo.co.jp

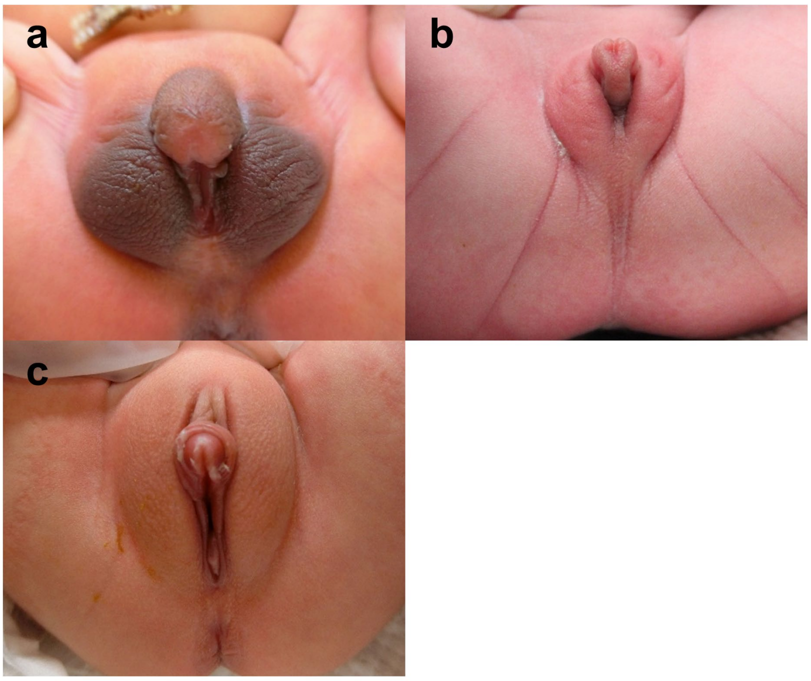


**Supplemental Figure.** External genitalia of patients with a diagnosis other than clitoral prepuce edema: a) a patient with 21-hydroxylase deficiency at age 5 days; b) a patient with persistent cloaca at birth; c) a patient with idiopathic clitoromegaly at age 103 days
